# Evaluating antimicrobial prescriptions in primary health care across an entire Brazilian city through the analysis of electronic medical records: where public health and data science converge

**DOI:** 10.1186/s12911-025-03260-9

**Published:** 2025-11-12

**Authors:** Ana R. C. Maita, Marcio K. Oikawa, Vítor Falcão de Oliveira, Viviane Aparecida Marto do Prado, Robson Pereira, Gabriela T. O. Xavier, Maria Laura Mariano de Matos, Erika Regina Manuli, Lucia H. A. R. Salvi, Monica Tilli Reis Pessoa Conde, Maria Clara Padoveze, Maria Tereza Razzolini, Nazareno Scaccia, Maura Salaroli de Oliveira, Ícaro Boszczowski, Cibele Cristine Remondes Sequeira, Regina Maura Zetone Graspan, Fabio Eudes Leal, Ester Cerdeira Sabino, Alison Holmes, Silvia Figueiredo Costa, Anna S. Levin, Fátima L. S. Nunes

**Affiliations:** 1 School of Arts, Sciences and Humanities, University of Sao Paulo, Sao Paulo, Brazil; 2 Departamento de Pesquisa Clínica e Inovação em Saúde, Universidade Municipal de São Caetano do Sul, Sao Paulo, Brazil; 3 Department of Infectious Diseases and Tropical Medicine, Institute of Tropical Medicine, Faculdade de Medicina, Universidade de Sao Paulo, Sao Paulo, Brazil; 4 School of Public Health, Universidade de São Paulo, Sao Paulo, Brazil; 5 Municipal Health Department, Primary Health System, Sao Caetano do Sul, Sao Paulo, Brazil; 6 Division of Infectious Diseases, Hospital das Clínicas, Faculdade de Medicina, Universidade de São Paulo, São Paulo, Brazil; 7 Department of Collective Health Nursing, School of Nursing, Universidade de Sao Paulo, Sao Paulo, Brazil; 8 University of Liverpool and Imperial College London, London, UK; 9 Divisão de Pesquisa Clínica (DIPETEC), Instituto Nacional do Câncer, Rio de Janeiro, Brazil; 10 Infection Control Department, Faculdade de Medicina, Hospital das Clínicas, Universidade de Sao Paulo, Sao Paulo, Brazil; 11 Information Technology Department of the Municipality of São Caetano do Sul, Sao Paulo, Brazil

**Keywords:** Antimicrobial prescription, Data science, Electronic medical records, Intervention

## Abstract

**Background:**

Exploring records from entire cities to make decisions, particularly within public health systems, remains challenging.

**Methods:**

This study investigates the public health data of São Caetano do Sul (SCS), in Brazil, to uncover patterns of antimicrobial prescriptions for infectious diseases using electronic health system records from primary care. Data science techniques such as preprocessing, transformation, loading, and analytics were also applied to achieve this goal.

**Results:**

From January to September 2023, a total of 575,616 records of medical appointments were analyzed, and 67,023 patients underwent one or more medical appointments of which 16,572 had infectious diagnoses. There were 7,938 prescriptions of antimicrobials for infections of which the most frequent were upper respiratory infections (37%), gingivitis/periodontal disease (20%), and urinary tract infections (9%). The most frequently prescribed antimicrobials were amoxicillin (23%), azithromycin (15%), amoxicillin/clavulanate (13%), ciprofloxacin (11%), and cephalexin (11%). A preliminary evaluation of the data highlighted several points for targeted interventions, as well as challenges in obtaining certain information. For instance, some infections lacked documented antimicrobial treatment, while others were managed with medications not considered first-line options.

**Conclusion:**

Implementing a system that can extract data directly from electronic records and automatically present it in a logical and relevant way to health professionals—including policymakers and administrators—would enable the identification of potential problems, the planning of interventions to improve antimicrobial use, and the monitoring of their impact. Our findings highlight opportunities to improve antimicrobial prescribing through data-driven tracking, analysis, and feedback mechanisms.

**Supplementary Information:**

The online version contains supplementary material available at 10.1186/s12911-025-03260-9.

## Introduction

Antimicrobial resistance (AMR) is a serious global public health problem leading to ineffective treatment, prolonged illness, increase in health care costs, and increase in mortality rates [1]. Its impact on mortality shows a trend that varies substantially by location and is more pronounced in developing countries [2]. South Asia, Latin America, and the Caribbean are forecasted to have the highest all-age AMR mortality rates by 2050 [3]. The inappropriate use of antimicrobial drugs is a relevant cause of the AMR, which should be mitigated. Under this perspective, the CAMO-Net (Centers for Antimicrobial Optimization Network) (https://camonet.org/*)* project is a unique global research partnership aimed at addressing AMR and supporting antimicrobial optimization. The studies also consider specific epidemiological, cultural, structural, and economic factors, supported by innovation and by the implementation of strategies [4]. 

Electronic Medical Records (EMRs) are electronic systems for clinical patient data collection. These electronic data have been increasingly used by primary health care (PHC) institutions as an important tool to support surveillance, monitoring, and intervention [5, 6]. The reduction of inappropriate prescription of antimicrobial drugs as well as the decrease in use of excessively broad antimicrobial coverage may drastically bring down the burden of the AMR [2, 7]. EMRs may potentially play a crucial role in this scenario by promoting the improvement of antimicrobial drug prescription using decision-making tools. In fact, antimicrobial stewardship based on EMRs medical appointments can result in decreasing unnecessary antimicrobial prescriptions and employing narrow-spectrum antimicrobial drug, thus slowing the spread of antimicrobial resistance and minimizing secondary infections such as by *Clostridioides difficile.* [7]

Despite the increased use of EMRs in healthcare systems, most countries still do not have an interconnected information network. Furthermore, antimicrobial consumption data in EMRs are limited, especially in PHC. Therefore, the evaluation of antimicrobial prescriptions and patient records may be a useful tool for evaluating the impact of management and educational strategies. The differential of this study is to present the analysis of data of medical appointments and prescribed drugs from an entire public PHC system in a Brazilian city to identify inadequacies that could be the target of interventions. This work presents the current scenario, under data analysis context, of São Caetano do Sul (SCS) as a case study for developing a multidisciplinary health intervention model.

This study focuses on the public primary care system, which comprises 12 primary health units strategically distributed to provide primary care. These units offer medical appointments, nursing care, vaccination, basic dental care, chronic disease management, and health promotion. Our analysis is designed considering the following research question: Is it possible to extract data from EMRs to assess prescription patterns for infectious diagnoses in SCS?

To address this research question, the present study aims to identify and characterize the profile of medical appointments in SCS between January and September 2023. The primary objective is to assess antibiotic use in consultations that included at least one infectious disease diagnosis, with particular attention to the drugs prescribed in these cases. Additionally, we also investigate prescription of non-antibicrobial drugs for infectious diagnoses. To achieve this, we applied a series of descriptive and cross-tabulation analyses, which allowed us to explore the data from multiple perspectives and to uncover patterns that may inform future research and support evidence-based decision-making in primary health care.

## Materials and methods

This section details the approach undertaken to obtain and transform the data, thus ensuring the reliability and validity of the study’s findings. The next subsections explain the process of data selection and acquisition, the preprocessing steps, cleaning and transforming raw data into structured formats, the analytical techniques, and the validation procedures implemented to verify the robustness of the findings.

### Materials

This study uses the EMR database records from the public health system of the city of SCS, São Paulo state, Brazil. SCS is located in the capital metropolitan region. It covers an area of 15,331 km^2^, an approximate population of 165,655 people, with the population density of 10,805.23 inhabitants/km^2^ [8].

SCS is considered to have a relatively well-structured Health Network, which provides distinct and complementary care services to its population: primary care, specialized care, pre-hospital care, emergency, outpatient and hospital care, epidemiological, sanitary, zoonosis, and health surveillance. To optimize the health operation and coverage, SCS health services also support several special projects, such as the Family Health Program (FHP), the structure of services in the Basic Health Care Units (BHCUs) and specific units [9]. 

Additionally, SCS provides a fully implemented and comprehensive EMR, and the local government collaborated with the project by providing data and supporting the study. The EMR and data availability do not reflect a common scenario for most municipalities in Brazil, so that SCS is an ideal city for study. Furthermore, SCS is also part of the national public health care network, allowing for the assessment of the effectiveness of national health policies and comparing them with other Brazilian cities.

The ethical guidelines and data privacy regulations were strictly followed in this study throughout the entire process to safeguard patient confidentiality and anonymity. Confidential and sensitive information were removed, and the identification codes were anonymized. All sensitive data, when necessary, were handled in person within the data-holding units, with isolation of external network access. Local ethical committees have approved the project that strictly complies with Brazilian data protection regulations [10]. The general and specific analysis results will support intervention strategies with the local public administration, aiming at the improvement of the planning for health care coverage.

Each registered medical appointment required the doctor, after evaluating the patient, to include at least one disease or health condition using the International Classification of Diseases (ICD 10) [11] or the International Classification of Primary Care (ICPC-2).^12^ The research team requested the extraction of specific data to meet the needs of the study: all medical appointments, drugs and tests prescribed, as well as the drugs dispensed during the period from January to September 2023. This extraction was formally delivered to the research group respecting all ethical and confidentiality issues in the raw files format. The complete dataset had 3,520,547 records, presented in Table S1. This table shows the amount of data extracted.

In this study we used EMR medical appointments and prescribed drugs. Although these tables are part of the same electronic health care system, we received them in separate files (see Table S1), linked by the patient identifier. Despite our attempts to use all the information, this was not possible because the Laboratory Tests file contains only exam orders from the public network, and the results performed in external laboratories are not accessible. The data referred to 77,507 patients in public health services such as PHC units, hospitals, and other public health facilities. This represented approximately 47% of the SCS’ population. A total of 67,023 of these individuals had medical appointments in the primary health units (approximately 40% of the population), which is the number of patients considered in the present study.

To assist the data retrieval and eliminate redundancies, a relational database was composed from the previously presented files, as shown in Figure S1 and Appendix 1, where TAB_PATIENT is a table that stores personal data of all patients who underwent medical appointments; TAB_UNIT_HEALTH_CARE encompasses all health care units in SCS, including PHC units and other units such as hospitals; TAB_MEDICAL_APPOINTMENT and TAB_MEDICAL_DIAGNOSIS contain, respectively, the descriptive data from the medical appointments, including a description of the patient’s symptoms, and the set of diagnoses identified by the doctor. The doctor completes this information during each medical appointment in the electronic health system at SCS during the period analyzed. The table named TAB_DRUG stores all the drugs available in pharmacies with the original data, such as product name, concentration, and presentation unit. It also contains an external field that maps the product name with its main chemical compound and its classification as antimicrobial or non-antimicrobial. The table TAB_DRUG_PRESCRIBED stores the relationship between the pharmacy code of the drug and the medical appointment in which it was prescribed.

Finally, TAB_ICD_DIAGNOSIS and TAB_ICPC_DIAGNOSIS contain codes and names of both infectious and non-infectious diagnoses. These tables were composed from the public reference bases available of International Classification of Diseases (ICD-10) [11] and International Classification of Primary Care (ICPC-2) [12] to appropriately classify diseases.

### Methods

**Fig. 1:**
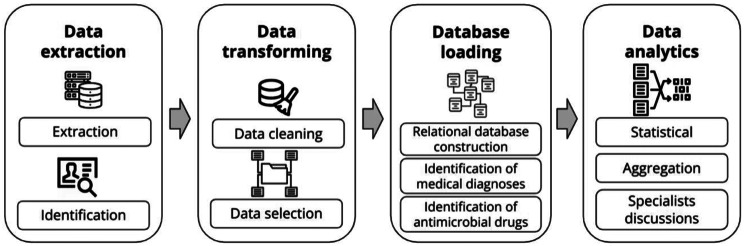
Stages of the information extraction procedure from public health data and steps carried out for each of them

This study follows the stages of knowledge discovery in the database (KDD) proposed by Han et al. [13] Since this is an initial study, we focus on the first stages (selection, preprocessing, and transformation) to guarantee the quality and reliability of the analyses. Figure 1 shows a summary of the developed stages, indicating the steps that composed each stage.

First, the **data extraction stage** involves receiving the data files in plain text format (as shown in Table S1) and understanding all field semantics. Then, the **transformation stage** executes data cleaning procedures to correct inconsistencies, and the selection of the most relevant information. Subsequently, in the **database loading stage**, we build a relational database. Additionally, we augmented the database with fields specifically designed to identify infectious and non-infectious diagnoses, as well as antimicrobial drugs (fields marked with an asterisk (*) in Figure S1). Finally, in the fourth **stage of data analytics**, several analyses of the data are undertaken with close collaboration with medical and health specialists to ensure their clinical relevance and accuracy. These analyses encompass both statistical and aggregation techniques. This sequential approach ensures a systematic and robust analysis grounded in both methodological rigor and expert domain knowledge, as shown in the next sections.

All processing tasks are carried out automatically and follow the appropriate programming standards to lay the foundations for a permanent technology environment. In this way, we apply technological tools and programming languages commonly used in data science. In the data loading stage, we created processing programs for relational databases by using Structured Query Language (SQL) of MySQL 8 Database Management System [14]. For data analysis, we used Python 3.10 [15] and the Plotly express [16] library, which promotes the manipulation of data and the creation of graphs for visualization of results.

### Data extraction

The EMR exports its internal data as CSV-formatted flat files, as a first step. An exploratory analysis was conducted to describe each field and file. During this stage, several discussions were held with both the Information Technology Office from SCS and physicians regarding the information recording process in the healthcare system, particularly concerning PHC.

Both the data extraction and analysis processes were conducted according to the Brazilian guidelines outlined [10]. The extracted flat files were not in an explicit relational structure. Thus, it was necessary to identify the relational pattern implicit in them. Because of this, the second step was the identification of entities related to the new relational database, such as health units, patient identification, and medical appointments. Fields that required external treatment were also identified to complement the information for interpretation, such as externally retrieving the name of the diagnosis using the ICD-10 or ICPC codes recorded for the medical appointment. Then, the primary and foreign keys were identified for each entity. In this way, the bases could be established for the stages described in the following sections.

### Data transforming

The files regarding medical appointments and prescribed drugs were processed in two steps: data cleaning and data selection. In the data cleaning step, the same procedures were applied to both files. First, all records in the table that did not have patient identifiers were removed. Next, we performed an analysis of missing data, focusing on empty identifiers or possible registration mistakes. Specifically in the medical appointments table, our attention was focused on the medical appointment identifier, and the ICD-10 or ICPC-2 disease identifiers, considered essential to this study. For the prescribed drugs, records that were not associated with any medical appointment were removed, either because they were out of the established period or due to some acquisition error. Next, all duplicate records in both tables were eliminated, as they did not provide additional information to the analysis.

**Fig. 2:**
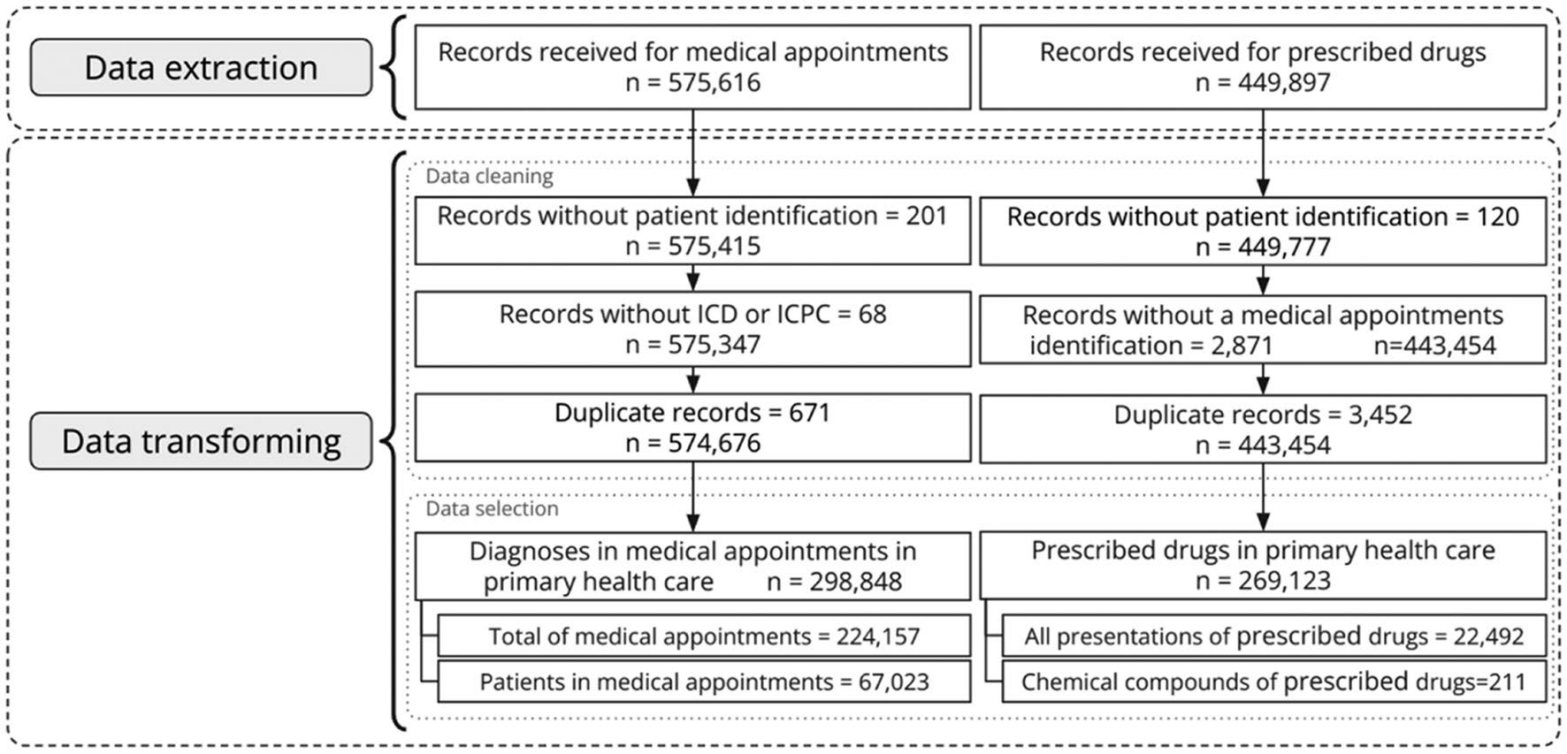
Total number of records in each step of the two first preprocessing stages (data extraction and data transforming)

Each step was carried out sequentially, as shown in Fig. 2, where the number of records resulting in each step is presented until reaching the final dataset used in the next stage. In the data selection step, the selection criterion is to preserve only PHC records as they had been previously established by the team of specialists. Figure 2 shows the final number of diagnoses. This number is greater than the medical appointments because the doctor might register one or more diagnoses for the same patient.

After the preprocessing stages, we identified a limitation in the analysis due to a characteristic of the EMR, in which each medical appointment can have several associated diagnoses and several prescribed drugs with the same appointment identifier, without establishing a direct relationship between a diagnosis and its prescribed drugs. So, in cases of medical appointments with more than one diagnosis, we could not accurately associate the prescribed drug with its corresponding diagnosis.

### Database loading

The preprocessing stages previously presented prepared the data to be included in the relational database model. This process involved reverse engineering to obtain a relational model, ensuring compliance with primary key constraints and respecting foreign key relationships throughout the data mapping effort (Figure S1). Then, the records from each file were analyzed and mapped to the corresponding relational table.

Additionally, in the data loading phase, the mapping of antimicrobial drugs was conducted using a keyword in the prescribed drug name record. This enables the grouping of drugs based on the principle of their main chemical compound. We excluded all drugs indicated for topical use, such as creams, eye drops, and sprays.

Table S2 presents the number of records in the files used in this study after the transformation stage. These numbers were extracted using aggregate queries on the relational database. The proposed structure allowed these calculations to be carried out in a more intuitive way while providing coherence between the related fields.

### Data analytics

Two integrated datasets were constructed: the first one contains diagnostic data at the medical appointments, and the second one contains medications prescribed at each medical appointment. These datasets were labeled by the type of diagnosis and the type of medication prescribed, respectively. Thus, diagnoses were categorized into infectious and non-infectious, while prescribed drugs were categorized into antimicrobial and non-antimicrobial. For this study, the analyzes were carried out based on the infectious diagnoses category of the diagnostic dataset.

**Fig. 3:**
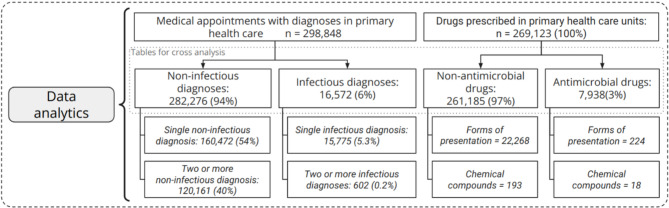
Data segmentation and number of records in pivot tables used in the analysis

As depicted in the diagram of Fig. 3, segregating the data by type for analysis promotes cross-referencing between tables on both sides. In this scenario, specifically, we made an exception to allow duplicates. Thus, single appointments with two (or more) diagnoses could have originated from two (or more) separate medical appointments because each different diagnosis might associate different prescribed drugs. These exceptional cases involving two or more infectious disease diagnoses accounted for only 0.2% of all medical appointment records, as shown in Fig. 3.

Finally, it was concluded that these duplicate data do not have a significant influence on the final analysis due to two aspects: *(i)* the low percentage of their occurrence, and *(ii)* most of these duplicate diagnoses refer to different organs of the human body (for example, respiratory system and urinary tract), but with an indication of infection in both.

### Exploratory case study

After discussing our results, we manually evaluated 40 patient records in which we observed a discrepancy between the recorded infectious diagnosis and its management. This was done to understand the context and the factors leading to the discrepancies. We selected a convenience sample, 20 with the diagnosis of syphilis and 20 with cystitis, the most commonly found discrepancies. These were reviewed by 3 infectious diseases physicians, searching for the information in the text of the EMRs, involving the open fields of the records, allowing a deeper analysis, as shown in the next section.

### Statistical analysis

Patients were stratified by infection site. We subsequently compared those who received antibiotics and those who did not, considering age and sex. We performed the Mann-Whitney U test for continuous variables and Fisher’s exact test for categorical variables. A p-value < 0.05 was considered statistically significant.

## Results

This section presents the results of the analyses encompassing the coverage of the data on the city’s population (Subsection 3.1); the frequencies of occurrences of infectious and non-infectious diagnoses, as well as the prescribed drugs classified as antimicrobial and non-antimicrobial (Subsection 3.2); a cross-reference of antimicrobial use in infectious diagnoses (Subsection 3.3); and the exploratory case study analyzing texts in the patient records (Subsection 3.4).

### Data coverage

The analysis of the population comprises patients who attended at least one medical appointment at a PHC unit in the city of SCS. These patients ranged in age from 0 to 101 years old. Diagnoses from the medical appointments were classified based on the presence or absence of an infectious diagnosis. In addition, these medical appointments were classified according to the prescription of antimicrobial or non-antimicrobial drugs. Table S3 presents the total records for the three age groups in a comparative manner, highlighting the types of diagnoses they received during medical appointments. This data stratification is used in the next section to determine the frequency and data crossing results.

### Most common diagnoses and prescribed drugs

**Fig. 4:**
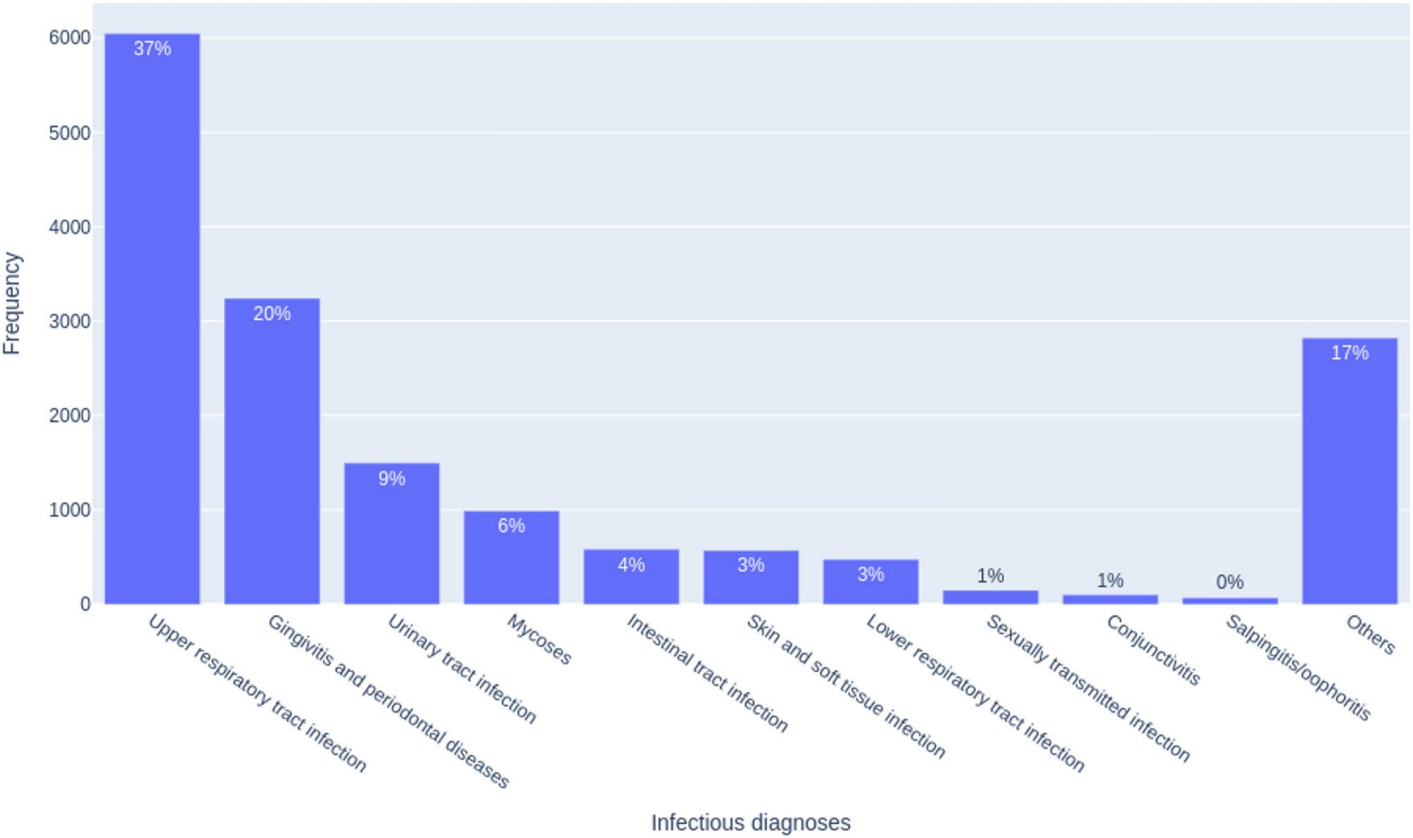
Frequency of the most common infectious diagnoses recorded during medical appointments in primary health care in the city of São Caetano do Sul (Brazil), January to September 2023

This section presents the analysis of frequencies, focusing on the classification of diagnoses and prescribed drugs. We examined the frequency of prescribed drugs and diagnoses encountered during medical appointments in PHC. From the relational model, we highlighted the distributions of infectious and non-infectious diagnoses recorded in the medical appointments in PHC, offering insights into prevalent health concerns encountered in these settings (Figs. 4 and S2). A total of 575,616 records of medical appointments were analyzed (Fig. 2), and 67,023 patients underwent one or more medical appointments of which 16,572 had infectious diagnoses (Table S3). There were 7,938 prescriptions of antimicrobials (Table S2 and Fig. 3). The most frequent infections were upper respiratory infections (37%), gingivitis/periodontal disease (20%), and urinary tract infections (9%) (Fig. 4), whereas the most prevalent non-infectious diagnosis was primary hypertension (10%) (Figure S2).

To better understand the characteristics of patients diagnosed with infections, a detailed categorization by age was conducted considering the ten most frequent infectious diagnoses. This analysis is depicted in Figure S3, where the bars indicate the percentage of diagnoses for each group concerning the total of medical appointments with infectious diagnosis. We observed a predominance of infectious diagnoses in adults, except for urinary tract infections, which were more common in the elderly.

Additionally, Figures S4 and Figure S5 show the frequency distributions of antimicrobial and non-antimicrobial drugs prescribed, respectively, providing insights into prescribing patterns within the context of primary health care. The most prescribed antimicrobials were amoxicillin (23%), azithromycin (15%), and amoxicillin-clavulanate (13%) (Figure S4), which align with the predominant diagnoses of upper respiratory tract infections. Among non-antimicrobial drugs, the most frequently prescribed were analgesics such as dipyrone (or metamizole) (5%) and medications belonging to the class of angiotensin receptor blockers (5%) (Figure S5).

### Cross analysis for medical appointments

In this analytical exploration, we conducted a data crossing to verify relationships between diagnoses and prescribed drugs, the resulting numbers are detailed in Table S4. To build this table, we cross-referenced diagnoses with prescribed medications. To do this, we took the infectious and non-infectious diagnosis datasets (see the “Type of diagnosis” column), then cross-referenced the antimicrobial and non-antimicrobial prescription datasets (see the “Prescribed medications for diagnoses column”). In the rows of this column, we put “All diagnoses” to refer to the entire table without cross-reference and “No medication prescription” when there was no prescribed medication during the medical appointment. In the “Number of records” column are the number of medical appointments with diagnoses in which no medication was prescribed, in which antimicrobials were prescribed, and in which other non-antimicrobial medications were prescribed. The last column “Number of records in cross-reference” contains the total records. When cross-referenced, this number can increase a lot because several medications may be prescribed for each medical appointment. For example, the number of records of medical appointments with a diagnosis of infection combined with the prescription of non-antimicrobial drugs increases almost threefold, since it is more common to prescribe several drugs of this type. We observed a higher proportion of medical appointments with an infectious diagnosis but without an antimicrobial prescription.

**Fig. 5:**
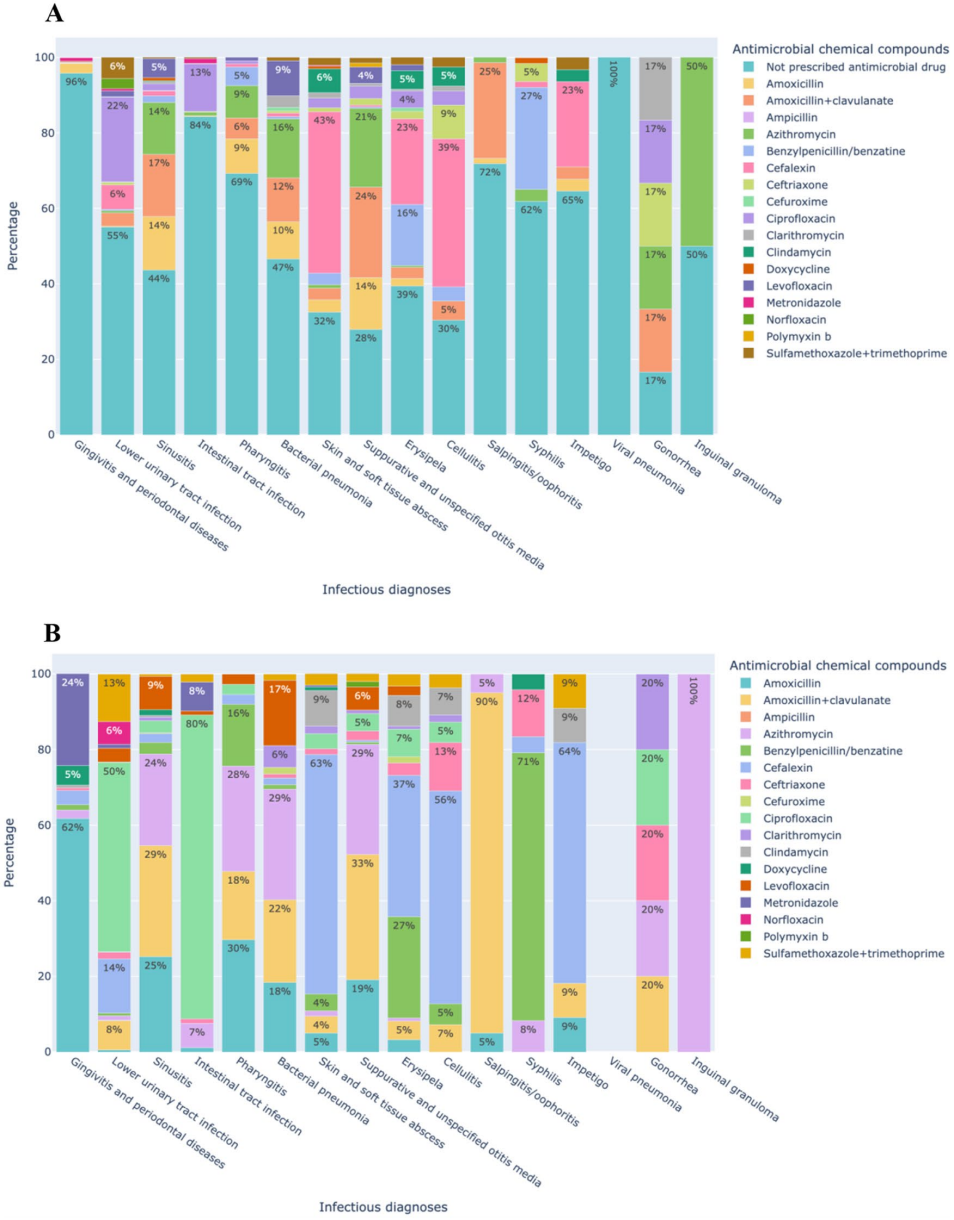
Distribution of treatments prescribed during medical appointments for infectious diagnosis in primary health care in the city of São Caetano do Sul (Brazil), January to September 2023. (**A**) All prescriptions. (**B**) Antimicrobial prescriptions

In many infectious conditions, no antimicrobials were prescribed, likely because the physician suspected a viral infection, especially in respiratory and intestinal infections (Fig. 5A). No antimicrobials were prescribed for 96% of gingivitis/periodontal disease, 84% of intestinal infections, and 69% of pharyngitis. Surprisingly, 55% of appointments in which lower urinary tract infection (which includes diagnoses such as cystitis) was registered and 62% of those with syphilis also did not register antimicrobial prescriptions.

Additionally, Fig. 5B presents the antimicrobial drugs prescribed for each of the infectious diagnoses in analysis present in the medical appointments. In this latter figure, we excluded non-antimicrobial prescribed drugs. Furthermore, to improve the visualization, we considered each diagnosis as 100% independently of its frequency. Thus, the percentages indicated in each bar correspond to the occurrences within the prescriptions for that specific diagnosis. For cases of gingivitis and periodontal disease, the two most commonly prescribed antimicrobials were amoxicillin (62%) and metronidazole (24%). In contrast, for lower urinary tract infections, ciprofloxacin (50%) was the most frequently prescribed antimicrobial (Fig. 5B). Furthermore, benzylpenicillin was chosen for bacterial pneumonia and erysipelas/cellulitis. Additionally, there were 5,376 (2%) medical consultations in which an antimicrobial drug was prescribed without a register of an infectious diagnosis. Many non-antimicrobial drugs were prescribed during medical appointments for infectious diagnoses, particularly symptomatic medications such as analgesics, anti-inflammatory drugs, and antihistamines (Table S5).

### Exploratory case study

We found that 17 out of 20 patients with syphilis had been treated with benzylpenicillin and were adequately followed-up. During the follow-up appointments the diagnosis was repeated but the antibiotic obviously was not prescribed again, thus creating the impression that the infection had not been treated. Of the remaining three diagnoses of syphilis, two were referred to other facilities outside of primary health care and one was interpreted by the doctor as having already been treated, requiring only follow-up.

In the cases of cystitis, we identified a variety of reasons for the discrepancies: in four cases, the physician did not prescribe antimicrobials despite registering a diagnosis of cystitis; in one case the patient was transferred to a hospital due to sepsis without receiving an antimicrobial prescription; three appointments were not adequately recorded in the EMR; in one case the doctor prescribed the antimicrobial treatment on the following day; in two cases the antimicrobial prescription was registered in the wrong section; and in one patient the antibiotic was changed due to clinical failure.

### Antibiotic use vs. non-use by infectious syndrome

For urinary tract infections, older patients received significantly fewer antibiotics (*p* < 0.001), whereas the opposite was observed for lower respiratory tract infections, where younger patients were prescribed fewer antibiotics (*p* < 0.001) (Table S6). For upper respiratory tract infections, women received more antibiotics (*p* = 0.009), while for gastrointestinal infections, men received significantly more antibiotics (*p* = 0.016).

## Discussion

Our analysis provides an overview of prescription practices for infectious diagnoses, focusing on a descriptive and comparative approach to real-world data. It assesses current practices, identifies intervention targets to improve antimicrobial prescribing and potentially reduce AMR. Additionally, it offers insights for healthcare decision-making and resource allocation, highlighting the role of data science in supporting intervention strategies in PHC.

Hospitals are more commonly the focus of studies evaluating inadequate antimicrobial treatment [17]. A study from Spain found that nearly 50% of prescriptions were inappropriate in the university hospital, influenced by factors such as age, empirical treatment, and infections of unknown or urinary site [18]. This inadequacy may be even more significant in PHC settings, where the bulk of antimicrobial prescriptions occurs [19]. Shively et al. evaluated the inadequacy of antimicrobial prescriptions and reported even higher rates, 76% of the reviewed prescriptions being deemed inappropriate [20]. Key factors contributing to inappropriate antimicrobial prescribing included comorbidities, the belief that PHC is not responsible for the development of antimicrobial resistance, and general practitioners’ perception that patients expect antimicrobials [21]. 

A preliminary analysis of our results allowed us to observe particularities in drug prescribing in the city. Amoxicillin was by far the most frequently prescribed antimicrobial drug. Other very frequently prescribed drugs were azithromycin, amoxicillin/clavulanate, doxycycline, cefalexin, and ciprofloxacin. Mujaini et al. reported that amoxicillin and cephalexin accounting for 46.2% and 21.7%, respectively, of all antibiotic prescriptions [22]. Consistently, amoxicillin was the most frequently prescribed antibiotic for suspected respiratory infections across South American countries [23]. 

Moreover, it is thought-provoking that no antimicrobial drugs were prescribed for a significant proportion of bacterial infections, such as cystitis and syphilis, despite the existence of clear guidelines for prescribing or withholding antimicrobials. This observation prompted our group to conduct an explanatory case study focusing on syphilis and uncomplicated lower urinary tract infections. For syphilis, we noted that the corresponding ICD code was routinely recorded for every follow-up medical appointment after treatment, causing the impression that the infection was not being treated. For cystitis, there were various explanations that pointed to areas in which improvement is warranted, including the choice of drug, and reinforcing the differences between infection and asymptomatic bacteriuria.

Additionally, there were instances where antimicrobial drugs were prescribed without an associated infectious diagnosis being documented. It is challenging to determine whether these cases represent antimicrobial misuse or errors in the recording of patient data. Nearly one-third of all prescriptions lacked documented clinical justification in PHC in England [24]. While improving antibiotic use was seen as a priority by Latin American healthcare workers, antibiotic use and AMR were not perceived as pressing concerns within their own facilities [25]. 

Another unexpected observation is the prescription of ceftriaxone in the PHC setting (2% of the total antimicrobial prescriptions). It was used mainly for gonorrhea because there have been no records of resistance to ceftriaxone per *Neisseria gonorrhoeae* in Brazil. However, it was also prescribed for syphilis. Other uses for this drug were cellulitis, erysipelas, and otitis media. It is challenging to explain this. These may have been complicated cases sent to primary care following a hospitalization or other complex health care. However, it points to the need for guidance and support from the primary care doctor or a specialist, especially in complex and worrisome clinical situations. A study showed that the most common inappropriate indications for ceftriaxone in the outpatient setting were cellulitis and non-severe pneumonia [26]. 

We evaluated the non-infectious diagnoses and the most frequently prescribed non-antimicrobial drugs. This was useful to evaluate the consistency and relevance of the database. A large part of medical appointments was due to routine evaluations. Essential hypertension, diabetes mellitus, and hypercholesterolemia were frequent diagnoses. In agreement with this, the most frequently prescribed non-antimicrobial drugs were symptomatic such as antipyretic drugs, antihypertensive drugs, antidiabetic drugs such as metformin, and cholesterol-lowering drugs such as statins.

The technical limitations of the EMR posed significant challenges to correlating diagnoses with prescribed drugs. In the collected data, a single medical appointment can encompass multiple diagnoses and several prescribed drugs. As already pointed out, it is not possible to precisely detect which prescribed drug was prescribed for which diagnosis. However, in the vast majority of cases with multiple diagnoses, only one infectious disease was identified, indicating minimal influence on our analysis. The next step is to apply data science methods to evaluate the appropriateness of antibiotic use. As elucidated in the data transformation section, this required broader analysis and the establishment of rules for selecting infectious diagnoses. Constraints within data capture systems may hinder the ability to conduct deeper analysis, thereby impeding the derivation of specific conclusions regarding healthcare practices.

Another limitation was how the drugs were recorded in the system, sometimes using their commercial name instead of their main chemical compositions. This made it challenging to group drugs, requiring computational and manual effort. These limitations may prevent the derivation of specific conclusions about real-world health care practices. Lastly, most diagnoses for major infectious syndromes, such as sexually transmitted infections, intestinal infections, and respiratory infections, were made empirically during the medical appointments due to the absence of specific diagnosis tests in PHC, specially in resource-limited settings such as Brazil. Furthermore, there is no systematic linkage between laboratory data and diagnostic information across different EMRs, as these data originate from more restricted and privacy-sensitive sources.

A system in place that can extract data directly from electronic systems and automatically present them in a way that is logical and relevant to health professionals, including policy makers and administrators, makes it possible to identify potential problems, plan interventions to improve antimicrobial use, and follow-up their impact. One of the strengths of our work is the mutual interaction of a multidisciplinary team (data scientists and health professionals) that does not usually share a common language.

Our next steps include promoting a system of feedback and automating the identification of inadequate drug prescriptions for the most prevalent infections, including the choice to treat or not, the choice of drug, and dosage and duration of treatment. These strategies can create a structured approach to improving care by identifying gaps, reinforcing evidence-based practices, and enhancing healthcare providers’ performance.

## Conclusions

This paper presents a general analysis of medical appointments considering drug prescriptions for infectious diagnoses in a public health system, from data existent in patient electronic records. Although the limitations of the data recording posed some challenges in the analysis, it is possible to verify the richness of information when data are cleaned, transformed, and processed.

Unquestionably, implementing EMR systems brings several advantages for healthcare management and patients, such as maintaining patient histories, integrating multiple healthcare units, more efficient processes, cost reduction, and a greater capacity for comprehensive medical evaluation. These data provide many opportunities for scientific research and better quality in the individual follow-up of patients. This study allowed us to identify critical points in the electronic health system, such as the lack of structured connections with external data sources, the absence of adequate identifiers for diseases and medications that enable cross-analysis, and, most importantly, the way data are recorded during consultations, which creates a barrier between the physician’s diagnosis and prescription and what is actually represented in the stored system data. Input into electronic records must follow clear standards and structured formats to ensure that information can be efficiently processed and interpreted. This approach would enhance transparency in antibiotic use and support the formulation of more objective interventions.

The analysis of SCS EMR provides insights into the current state of the public PHC system, highlighting its key capabilities, characteristics, strengths, and weaknesses. Despite the substantial availability of data, processing, interpreting, and analyzing them remains challenging. Our findings indicate prescription rates recorded in the digital system that appear suboptimal; however, these rates do not necessarily reflect the actual clinical reality. This observation highlights numerous opportunities to improve how electronic systems capture data, enabling them to reveal more accurate and detailed patterns of medication prescriptions at the moment of the physician’s diagnosis. When integrated into health systems, data science supports these efforts by identifying gaps in care, informing clinical and policy decisions, and enhancing surveillance through the transformation of large-scale health data into actionable insights. Recognizing this limitation and the lessons learned from this study provides insights to improve data quality and guide more effective health information systems, offering an important opportunity to strengthen antimicrobial stewardship and inform public health strategies for improvement within PHC.

## Supplementary Information

Below is the link to the electronic supplementary material.

Supplementary Material 1

## Data Availability

Data is provided within the manuscript or supplementary information files.

## Declarations

### Relevant guidelines and regulations

Local ethical committees have approved the project that strictly complies with Brazilian data protection regulations, specifically L. 13709/18 (General Data Protection Law) and CNS Res. 738/24 (regulates the use of databases for health scientific research involving human subjects).
