# Supplementary material for: Evaluating antimicrobial prescriptions in primary health care across an entire Brazilian city through the analysis of electronic medical records: where public health and data science converge
Source: BMC Med Inform Decis Mak. 2025 Nov 12;25:421. doi: 10.1186/s12911-025-03260-9 (PMC12613338; doi:10.1186/s12911-025-03260-9)
Supplement: Supplementary file 1 — Supplementary Material 1 [file 12911_2025_3260_MOESM1_ESM.docx]

**Supplementary Material**

**Maita ARC *et al*.** Evaluating antimicrobial prescriptions in primary health care across an entire Brazilian city through the analysis of electronic medical records: where public health and data science converge

| Figure S1 | Page 2 |
| --- | --- |
| Figure S2 | Page 3 |
| Figure S3 | Page 4 |
| Figure S4 | Page 5 |
| Figure S5 | Page 6 |
| Table S1 | Page 7 |
| Table S2 | Page 8 |
| Table S3 | Page 9 |
| Table S4 | Page 10 |
| Table S5 | Pages 11-12 |
| Table S6 | Page 13 |
| Appendix 1 | Pages 14-15 |

**Figure S1.** Relational database diagram built from data available in the files received from EHS.


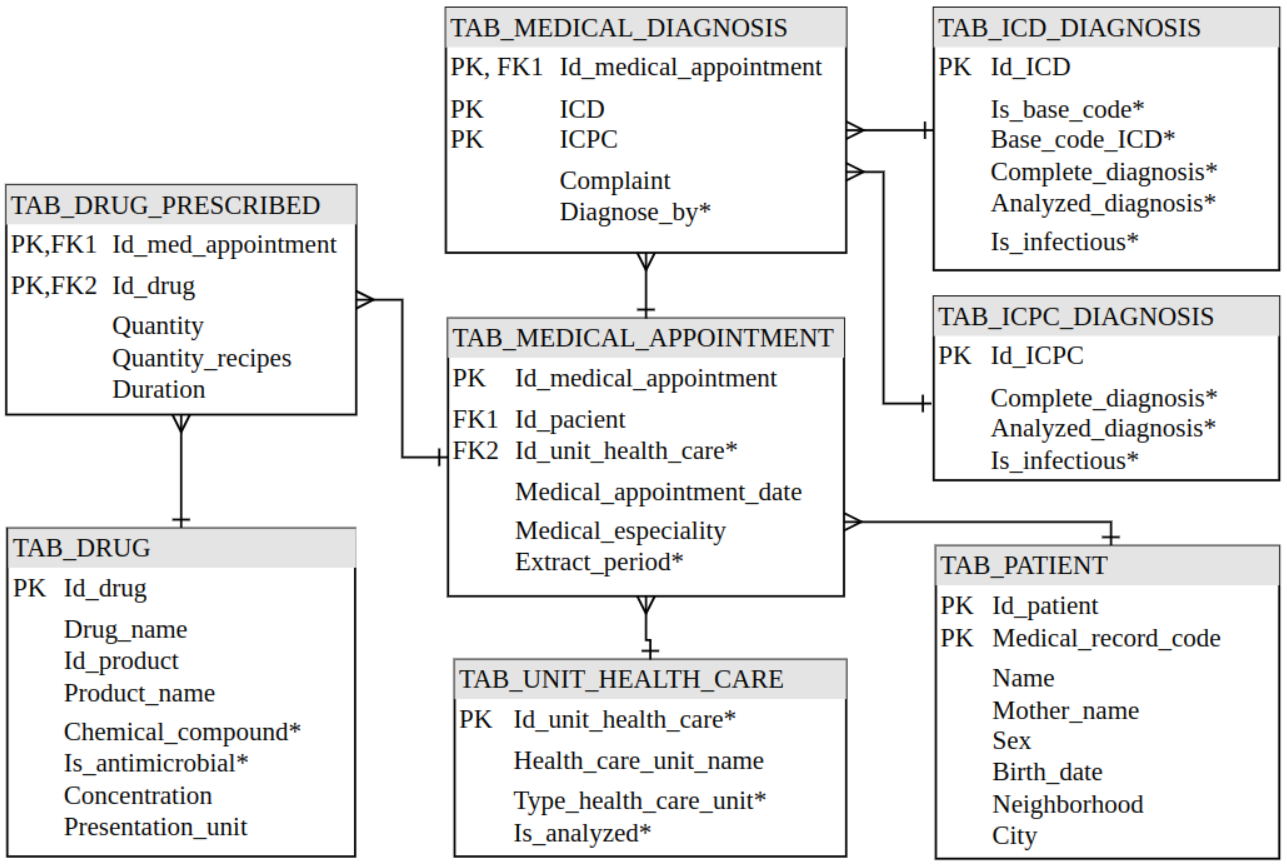


Some fields were included to complete the requirements of the analysis. These fields are indicated with an asterisk (*) next to their name in each table.

**Figure S2.** The 20 most common non-infectious diagnoses/conditions recorded in medical appointments in primary health care in the city of São Caetano do Sul (January-September 2023).


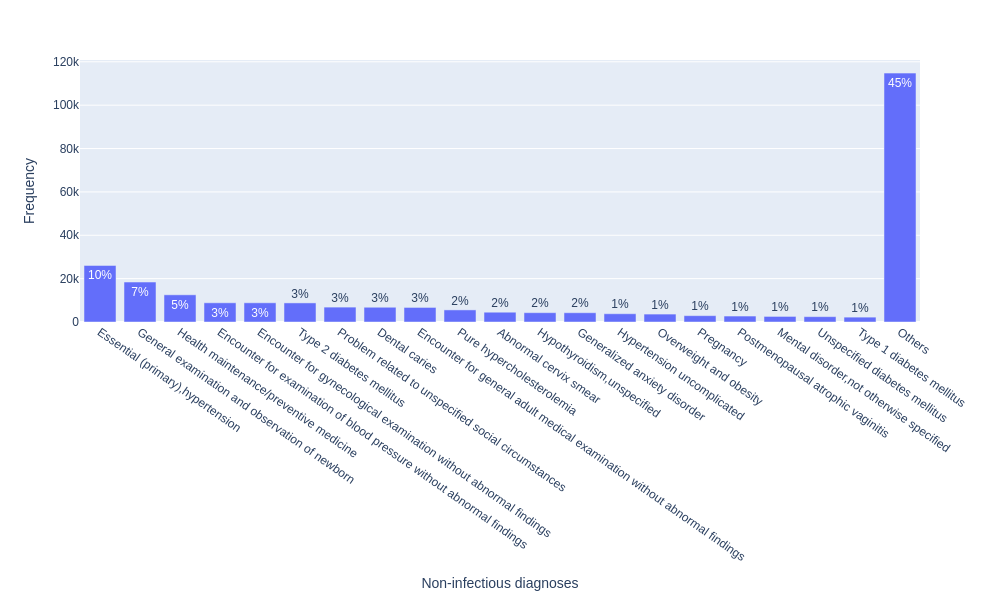


**Figure S3.** Frequency of infectious diagnoses at medical appointments by age group (São Caetano do Sul, Brazil. January-September 2023).


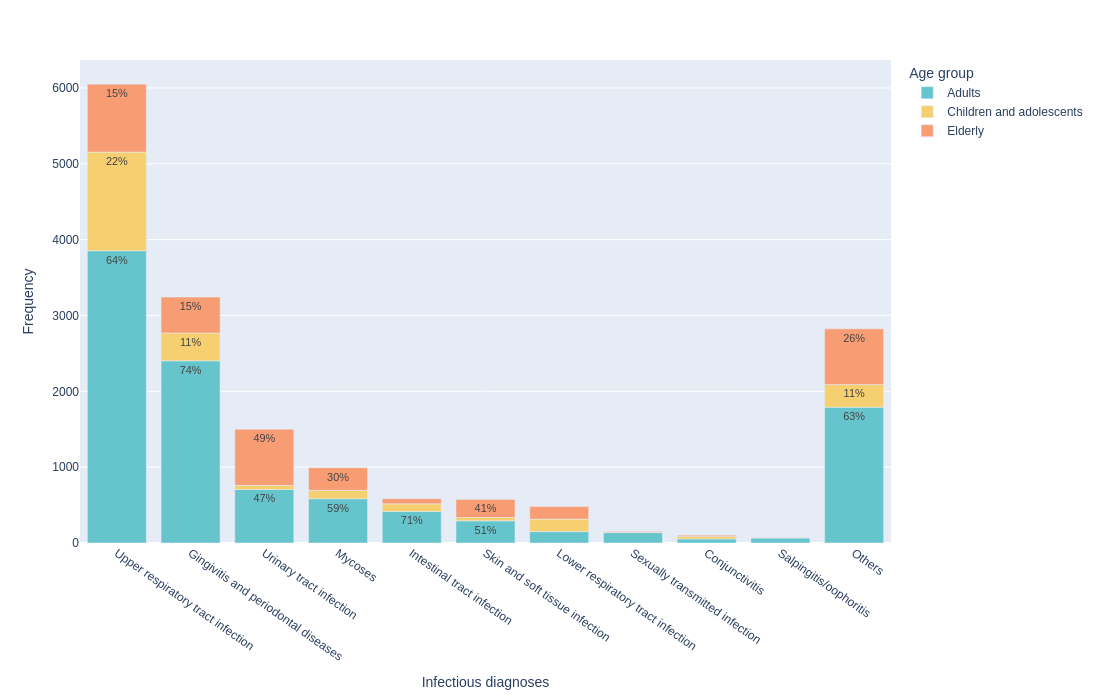


**Figure S4.** Frequency of prescribed antimicrobial drugs in medical appointments of primary health care in the city of São Caetano do Sul (January-September 2023).


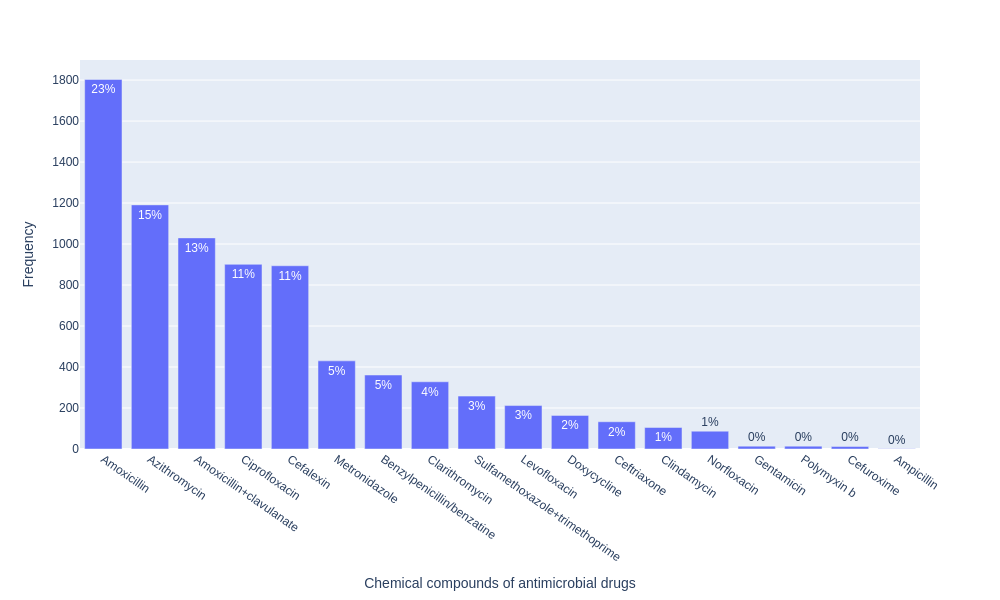


**Figure S5.** Frequency of prescribed non-antimicrobial drugs in medical appointments of primary health care in the city of SCS (January-September 2023).


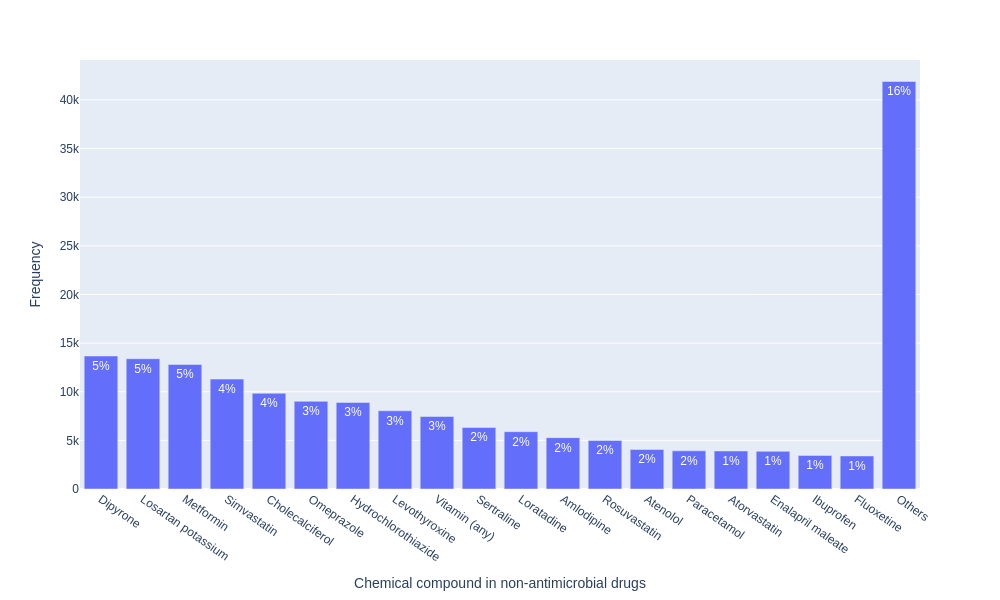


**Table S1.** Description of the files with medical records received from the public health system of the city of São Caetano do Sul in Brazil, corresponding to the period January-September 2023

| **File** | **Nr. of records** | **Description** |
| --- | --- | --- |
| Medical appointments | 575,616 | List of all medical appointments recorded in the SCS public health system. It includes descriptive data of patients, as well as all illnesses and diagnoses identified by the doctor. |
| Prescribed drugs | 449,897 | List of all prescribed drugs in the medical appointments, including descriptive information about the treatment. |
| Dispensed drugs | 1,055,152 | List of drugs that were dispensed by the pharmacies of the SCS public health system, including product information and number of units dispensed. |
| Laboratory tests | 1,439,882 | List of laboratory tests prescribed during appointments in the SCS public health system. |

**Table S2.** Number of records in the relational database after data transforming stage.

| **Data from relational database** | **Nr. of records** |
| --- | --- |
| Primary health care units | 12 |
| All ICD diagnoses used in search at medical appointments | 896 |
| Infectious diagnoses by ICD | 366 |
| Non-infectious diagnoses by ICD | 530 |
| All ICPC diagnoses used in search at medical appointments | 726 |
| Infectious diagnoses by ICPC | 78 |
| Non-infectious diagnoses by ICPC | 648 |
| Groups of common infectious diagnoses in medical appointments | 11 |
| Infectious diagnoses in medical appointments under analysis | 16 |
| Prescribed drugs | 269,123 |
| Antimicrobial drugs prescribed during medical appointments | 7,938 |
| Non-antimicrobial drugs prescribed during medical appointment | 261,185 |
| Medical appointments with prescribed drugs | 94,244 |
| Antimicrobial drugs (See Figure S4) | 7,115 |
| Non-antimicrobial drugs (See Table S5) | 87,129 |

**Table S3.** Patients and prescribed medications grouped by age group.

| **Data by age groups** | **Children & adolescents** | **Adults** | **Elderly** | **Total (100%)** |
| --- | --- | --- | --- | --- |
| Patients in primary health care | 10,615 (16%) | 37,268 (56%) | 19,140 (29%) | 67,023 |
| Medical appointments | 28,437 (10%) | 157,398 (53%) | 113,013 (38%) | 298,848 |
| Infectious | 2,480 (15%) | 10,451 (63%) | 3,641 (22%) | 16,572 |
| Non-infectious | 25,957 (9%) | 146,947 (52%) | 109,372 (39%) | 282,276 |
| Medical appointment with prescribed drugs | 8,031 (9%) | 49,984 (53%) | 36,229 (38%) | 94,244 |
| Antimicrobial drug | 647 (9%) | 4,601 (65%) | 1,867 (26%) | 7,115 |
| Non-antimicrobial drug | 7,384 (8%) | 45,383 (52%) | 34,362 (39%) | 87,129 |

**Table S4.** Cross-reference of the datasets of medical appointments with infectious and non-infectious diagnoses, examining the respective prescribed drugs (at least one antimicrobial or only non-antimicrobial) or cases with no drug prescription.

| **Type of diagnosis** | **Prescribed drugs for diagnoses** | **Nr. of records** | **Nr. records in cross-reference** |
| --- | --- | --- | --- |
| Infectious | All diagnoses (See Figures 4 and S3) | 16,572 (100%) | - |
| Infectious | No drug prescription | 6,188 (38%) | - |
| Infectious | Only non-antimicrobial drugs prescribed (See Table S5) | 6,762 (41%) | 18,467 |
| Infectious | At least one antimicrobial drug prescribed | 3,622 (22%) | - |
| Infectious | Diagnoses in analysis (See Figure 5) | 7,835 (47%) | 8,007 |
| Infectious | Antimicrobial drug prescribed for diagnoses in analysis (See Figure 6) | 2,179 (13%) | 2,351 |
| Non-infectious | All diagnoses (See Figure S2) | 282,276 (100%) | - |
| Non-infectious | No drug prescription | 149,158 (53%) | - |
| Non-infectious | Only non-antimicrobial drugs prescribed | 127,687 (45%) | - |
| Non-infectious | At least one antimicrobial drug prescribed | 5,431 (2%) | - |

**Table S5.** Summary table of non-antimicrobial medications prescribed during medical consultations with an infectious diagnosis.

| **Chemical compost** | **Diagnosis** | | | | | | | | | | |
| --- | --- | --- | --- | --- | --- | --- | --- | --- | --- | --- | --- |
|  | **Conjunctivitis**  **(n=121)** | **Gingivitis and periodontal diseases**  **(n=197)** | **Intestinal tract infection**  **(n=1424)** | **Lower respiratory tract infection**  **(n=318)** | **Mycoses**  **(n=1340)** | **Salpingitis / oophoritis**  **(n=62)** | **Sexually transmitted infection**  **(n=68)** | **Skin and soft tissue infection**  **(n=228)** | **Upper respira- tory tract infection**  **(n=9950)** | **Urinary tract infection**  **(n=995)** | **Others**  **(n=3764)** |
| Ambroxol | - | - | - | - | - | - | - | - | 645 (6.0%) | - | - |
| Diclofenac sodium | - | - | - | - | - | 18 (29.0%) | - | - | - | - | - |
| Dipyrone | 15 (12.0%) | 51 (26.0%) | 360 (25.0%) | 33 (10.0%) | - | - | 5 (7.0%) | 21 (9.0%) | 2,143 (22.0%) | 46 (5.0%) | 534 (14.0%) |
| Fluconazole | - | - | - | - | 242 (18.0%) | 3 (5.0%) | - | - | - | - | - |
| Ibuprofen | - | 19 (10.0%) | - | - | - | 4 (6.0%) | - | 11 (5.0%) | 727 (7.0%) | - | - |
| Ketoconazole | - | - | - | - | 67 (5.0%) | - | - | - | - | - | - |
| Loratadine | 21 (17.0%) | - | - | 19 (6.0%) | - | - | - | - | 1,753 (18.0%) | - | 428 (11.0%) |
| Metoclopramide hydrochloride | - | - | 105 (7.0%) | - | - | - | - | - | - | - | - |
| Nimesulide | - | 53 (27.0%) | - | - | - | 21 (34.0%) | - | - | 1,080 (11.0%) | - | - |
| Nitrofurantoin | - | - | - | - | - | - | - | - | - | 225 (23.0%) | - |
| Norethisterone | - | - | - | - | - | 3 (5.0%) | - | - | - | - | - |
| Nystatin | - | - | - | - | 92 (7.0%) | - | - | - | - | - | - |
| Omeprazole | - | - | 66 (5.0%) | - | - | - | - | - | - | - | - |
| Prednisolone | - | - | - | - | - | - | - | - | - | - | - |
| Prednisone | - | - | - | 26 (8.0%) | - | - | - | - | 681 (7.0%) | - | - |
| Rehydration salts | - | - | 153 (11.0%) | - | - | - | - | - | - | - | - |
| Salbutamol | - | - | - | 37 (12.0%) | - | - | - | - | - | - | - |
| Saline solution | 9 (7.0%) | - | - | - | - | - | - | - | - | - | - |
| Vitamins | - | - | - | 26 (8.0%) | - | - | 4 (6.0%) | - | - | - | - |
| Others non-antimicrobial drugs | 33 (27.0%) | 64 (32.0%) | 299 (21.0%) | 42 (13.0%) | 406 (30.0%) | 3 (5.0%) | 31 (46.0%) | 42 (18.0%) | 787 (8.0%) | 189 (19.0%) | 566 (15.0%) |
| Non-antimicrobial drugs (< 5%) | 43 (35.5%) | 10 (5.1%) | 441 (31.0%) | 135 (42.5%) | 533 (39.8%) | 10 (16.1%) | 28 (41.2%) | 154 (67.5%) | 2,134 (21.4%) | 535 (53.8%) | 2,236 (59.4%) |

**Table S6.** Comparison of patients groups with and without antibiotic prescriptions by infectious syndrome

| **Characteristics of patients** | **No antibiotic prescribed**  n (%) | **Antibiotic prescribed**  n (%) | **p-value** |
| --- | --- | --- | --- |
| **Urinary tract infection** | 831 | 643 |  |
| Female | 701 (84) | 529 (82) | 0.3 |
| Median age, years (IQR) | 66 (49 - 80) | 61 (46 - 74) | **< 0.001** |
| **Lower respiratory tract infection** | 301 | 165 |  |
| Female | 182 (60) | 104 (63) | 0.6 |
| Median age, years (IQR) | 27 (3 - 70) | 59 (42 - 72) | **< 0.001** |
| **Upper respiratory tract infection** | 4,038 | 1,715 |  |
| Female | 2,580 (64) | 1,157 (67) | **0.009** |
| Median age, years (IQR) | 41 (19 - 58) | 39 (23 - 55) | 0.3 |
| **Skin and soft tissue infection** | 224 | 337 |  |
| Female | 158 (71) | 211 (63) | 0.053 |
| Median age, years (IQR) | 62 (43 - 75) | 58 (44 - 75) | 0.7 |
| **Mycoses** | 931 | 34 |  |
| Female | 655 (70) | 26 (76) | 0.4 |
| Median age, years (IQR) | 50 (32 - 67) | 48 (31 - 63) | 0.6 |
| **Intestinal tract infection** | 485 | 85 |  |
| Female | 301 (62) | 41 (48) | **0.016** |
| Median age, years (IQR) | 34 (21 - 52) | 33 (24 - 45) | 0.9 |
| **Sexually transmitted infection** | 108 | 40 |  |
| Female | 57 (53) | 18 (45) | 0.4 |
| Median age, years (IQR) | 34 (26 - 51) | 31 (27 - 43) | 0.2 |

IQR: interquartile range

**Appendix 1.** Data dictionary of the fields used in the analysis. All fields of the relational database model are found in Figure S1.

| **Table/Field name** | **Field type** | **Description** |
| --- | --- | --- |
| **TABLE PATIENT** | | |
| Id_patient | String | Patient’s CPF (Brazilian person identification) |
| Medical_record_code | Integer | Record code in public health care system |
| Name, Mother_name, Sex | String | Patient’s personal data |
| Birth_date | Date | Patient’s date of birth |
| Neighborhood, city | String | Patient’s residence place |
| **TABLE MEDICAL APPOINTMENT** | | |
| Id_med_appointment | Integer | Medical appointment identification code |
| Health_care_date | Date | Date of the medical appointment |
| Medical_speciality | String | Specialist of the medical appointment |
| **TABLE MEDICAL DIAGNOSIS** | | |
| Id_ICD | String | International code for the disease/health cause |
| Id_ICPC | String | Brazilian code for the disease/health cause |
| Complaint | String | Patient complaint description |
| Diagnose_by | Bool | Flag to define which disease classification is used in the final diagnosis of medical appointment, this can be 0: ICD or 1: ICPC |
| **TABLE ICD DIAGNOSIS** | | |
| Id_ICD | String | International code for the disease/health cause |
| Is_base_code | Bool | Flag to define if the Id ICD, 1: represents a group of diagnosis or 0: if it is a particular disease |
| Complete_disease | String | Name of disease/health cause in English, taken from [(Anon n.d.)](https://sciwheel.com/work/citation?ids=16888318&pre=&suf=&sa=0&dbf=0) |
| Analyzed_disease | String | Short version of the name of ICD disease/medical appointment cause for analysis |
| Is_infectious | Boolean | Classification as infectious and non-infectious diagnosis |
| **TABLE ICPC DIAGNOSIS** | | |
| Id_ICPC | String | Brazilian code for the disease/health cause |
| Complete_disease_pt | String | Name of disease/health cause in English, taken from [(Anon n.d.).](https://sciwheel.com/work/citation?ids=16888324&pre=&suf=&sa=0&dbf=0) |
| Analyzed_disease | String | Short version of the name of ICPC disease/medical appointment cause for analysis |
| Is_infectious | Boolean | Classification as infectious and non-infectious diagnosis |
| **TABLE DRUG** | | |
| Id_drug | Integer | Identification of the drug in original health care system |
| Drug_name | String | Name of the drug in original health care system |
| Id_product | String | Identification of the product in original health care system |
| Product_name | String | Name of the product in original health care system |
| Chemical_compound | String | Chemical compound mapped externally |
| Is_antimicrobial | Boolean | Classification as antimicrobial and non-antimicrobial drugs |
| Concentration | String | Drug’s concentration in product |
| Presentation_unit | String | Product’s presentation unit |
| **TABLE PRESCRIBED DRUG** | | |
| Quantity | Integer | Quantity of prescribed drugs for in each recipe |
| Quantity_recipes | Integer | Quantity of recipes prescribed for the health care |
| Duration | String | Treatment duration |
| **TABLE UNIT HEALTH** | | |
| Id_unit_health_care | Integer | Health care unit’s identification |
| Unit_name | String | Health care unit’s name |
| Type_unit | String | Classification of health care unit |
| Is_analyzed | Boolean | Filter to consider health care unit in analysis |
